# Supplementary material for: West Nile Virus, an Underdiagnosed Cause of Acute Fever of Unknown Origin and Neurological Disease among Hospitalized Patients in South Africa
Source: Viruses. 2023 Nov 2;15(11):2207. doi: 10.3390/v15112207 (PMC10674603; doi:10.3390/v15112207)
Supplement: Supplementary file 1 [file viruses-15-02207-s001.zip › viruses-2664881-supplementary.pdf]

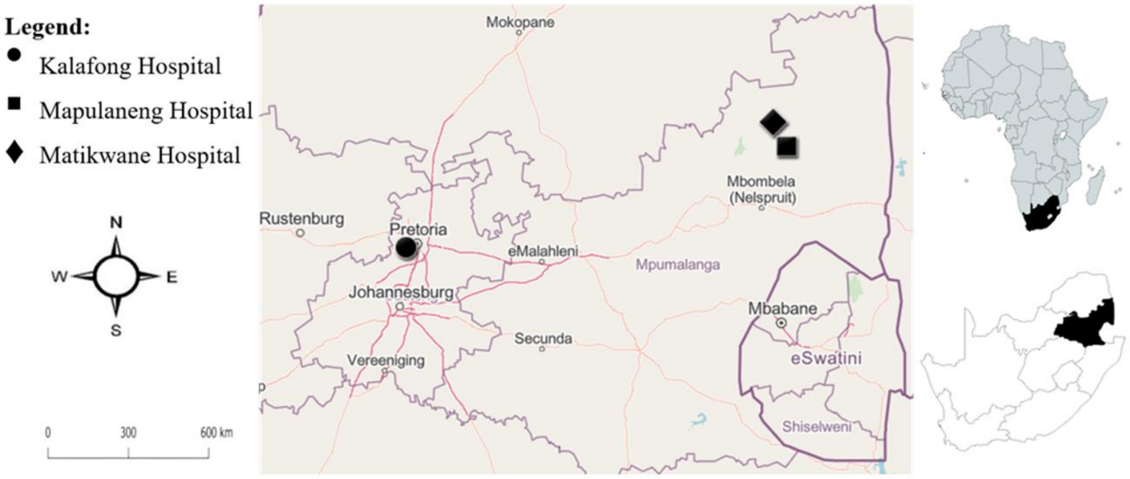

Figure S1: Map showing the locations of the 10 public sector hospitals where CSF was sampled from patients experiencing acute neurological disease in 2017 that made up the neurological cohort

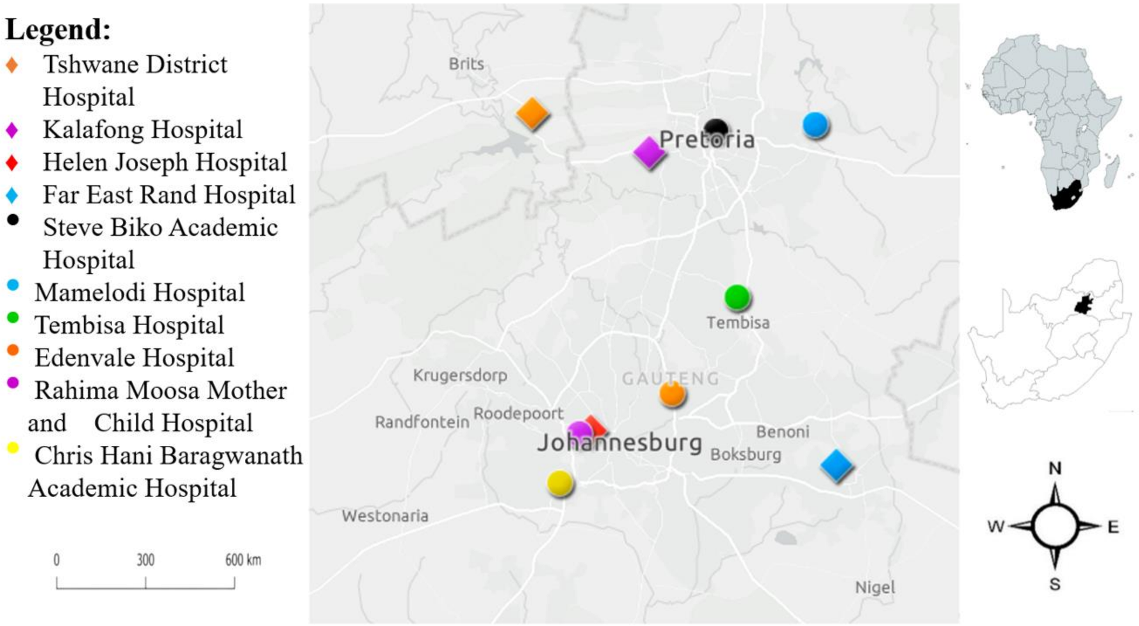

Figure S2: The three hospitals that make up the ANDEMIA sentinel sites. The urban Gauteng site comprised of Kalafong Provincial Tertiary Hospital and the rural Mpumalanga site, comprised of the Mapulaneng and Matikwane Hospitals,

Table S1. Details of the flavivirus isolates used in additional neutralization tests

**Virus**	**Isolate name, passage number**	**Details**	**GenBank Accession no.**
Banzi virus	LAP13MP25, P2	Isolate obtained from a homogenate of *Culex rubinotus* mosquitoes collected in 2013 in Lapalala, South Africa	OL411961
Bagaza virus	ZRU349/17/3, P5	Isolate obtained from the brain of a Himalayan monal pheasants (*Lophophorus impejanus*) who had presented with neurological signs	MN329586
Usutu virus	SAAr 1776, P7	Isolate first obtained 1958 from mosquitoes in South Africa. The isolate was supplied by the Centers for Disease Control and Prevention, Fort Collins, USA.	AY453412
Wesselsbron virus	AV259, P4	Isolate obtained from a non-fatal encephalitic human case. Supplied by the National Institute of Communicable Diseases, Gauteng, South Africa	JN226796

Table S2: Details of the PCR, ELISA and VNT results for the WNV positive patients detected in the neurological cohort in 2017 and in the AFDUC cohort, 2019 to 2021.

ZRU Number	Year enrolled	Hospital	Age (years)	Sex	Disease presentation	PCR	WNV IgM	WNV VNT	VNT titer	Specimen tested
**Neurological cohort**										
ZRUNH072/17	2017	Tembisa hospital	28	Female	Meningitis	Negative	Positive	Positive	≥1:32	CSF
ZRUNH212/17	2017	Mamelodi Regional Hospital	1	Female	Encephalitis	Negative	Positive	Positive	≥1:16	CSF
ZRUNH267/17	2017	Steve Biko Academic Hospital	62	Male	Encephalitis	Negative	Positive	Positive	≥1:32	CSF
ZRUNH273/17	2017	Mamelodi Regional Hospital	1	Female	Febrile convulsion	Positive	Negative	Positive	Not performed	CSF
ZRUNH299/17	2017	Steve Biko Academic Hospital	28	Female	Encephalopathy	Negative	Inconclusive	Positive	≥1:16	CSF
ZRUNH497/17	2017	Mamelodi Regional Hospital	2	Male	Meningitis	Positive	Negative	Positive	Not performed	CSF
ZRUNH511/17	2017	Kalafong Provincial Tertiary Hospital	19	Female	Meningitis	Negative	Positive	Positive	≥1:32	CSF
ZRUNH557/17	2017	Kalafong Provincial Tertiary Hospital	1	Female	Meningitis	Negative	Positive	Positive	≥1:16	CSF
**AFDUC cohort**										
ZRUA0336/19	2019	Kalafong Provincial Tertiary Hospital	1	Female	Severe	Negative	Positive	Positive	≥1:32	Plasma/Serum
ZRUA0360/19	2019	Kalafong Provincial Tertiary Hospital	2	Female	Moderate	Negative	Inconclusive	Positive	≥1:32	Plasma/Serum
ZRUA0378/19	2019	Mapulaneng Hospital	22	Male	Severe	Negative	Positive	Positive	≥1:32	Plasma/Serum
ZRUA0382/19	2019	Kalafong Provincial Tertiary Hospital	36	Male	Severe	Negative	Inconclusive	Positive	≥1:32	Plasma/Serum
ZRUA0394/19	2019	Mapulaneng Hospital	1	Male	Severe	Negative	Inconclusive	Positive	≥1:32	Plasma/Serum
ZRUA0398/19	2019	Matikwana Hospital	16	Female	Severe	Negative	Positive	Positive	≥1:32	Plasma/Serum

ZRUA0431/19	2019	Matikwana Hospital	43	Female	Severe	Negative	Inconclusive	Positive	≥1:32	Plasma/Serum
ZRUA0469/19	2019	Mapulaneng Hospital	34	Male	Severe	Negative	Inconclusive	Positive	≥1:32	Plasma/Serum
ZRUA0471/19	2019	Kalafong Provincial Tertiary Hospital	2	Female	Moderate	Negative	Positive	Positive	≥1:16	Plasma/Serum
ZRUA0502/19	2019	Mapulaneng Hospital	1	Male	Severe	Negative	Positive	Positive	≥1:32	Plasma/Serum
ZRUA0549/19	2019	Matikwana Hospital	13	Male	Severe	Negative	Positive	Positive	≥1:32	Plasma/Serum
ZRUA0573/19	2019	Kalafong Provincial Tertiary Hospital	1	Female	Severe	Negative	Positive	Positive	≥1:32	Plasma/Serum
ZRUA0576/19	2019	Kalafong Provincial Tertiary Hospital	4	Female	Moderate	Negative	Inconclusive	Positive	≥1:16	Plasma/Serum
ZRUA0580/19	2019	Matikwana Hospital	57	Female	Moderate	Negative	Inconclusive	Positive	≥1:32	Plasma/Serum
ZRUA0583/19	2019	Kalafong Provincial Tertiary Hospital	1	Female	Severe	Negative	Inconclusive	Positive	≥1:32	Plasma/Serum
ZRUA0637/19	2019	Kalafong Provincial Tertiary Hospital	0	Female	Severe	Negative	Inconclusive	Positive	≥1:32	Plasma/Serum
ZRUA1095/20	2020	Mapulaneng Hospital	8	Female	Severe	Negative	Positive	Positive	≥1:32	Plasma/Serum
ZRUA1117/20	2020	Mapulaneng Hospital	36	Male	Severe	Negative	Positive	Positive	≥1:16	CSF
ZRUA1120/20	2020	Mapulaneng Hospital	90	Male	Severe	Negative	Inconclusive	Positive	≥1:32	Plasma/Serum
ZRUA1208/20	2020	Kalafong Provincial Tertiary Hospital	8	Male	Moderate	Negative	Inconclusive	Positive	≥1:32	Plasma/Serum
ZRUA1231/20	2020	Kalafong Provincial Tertiary Hospital	2	Female	Severe	Negative	Inconclusive	Positive	≥1:32	Plasma/Serum
ZRUA1235/20	2020	Kalafong Provincial Tertiary Hospital	6	Female	Severe	Negative	Positive	Positive	≥1:32	Plasma/Serum
ZRUA1249/20	2020	Kalafong Provincial Tertiary Hospital	4	Male	Moderate	Negative	Inconclusive	Positive	≥1:32	Plasma/Serum
ZRUA1252/20	2020	Mapulaneng Hospital	23	Male	Moderate	Negative	Positive	Positive	≥1:32	Plasma/Serum
ZRUA1258/20	2020	Mapulaneng Hospital	21	Female	Severe	Negative	Positive	Positive	≥1:32	Plasma/Serum
ZRUA1265/20	2020	Kalafong Provincial Tertiary Hospital	0	Female	Severe	Negative	Positive	Positive	≥1:32	Plasma/Serum

ZRUA1329/20	2020	Kalafong Provincial Tertiary Hospital	1	Male	Moderate	Negative	Inconclusive	Positive	≥1:16	Plasma/Serum
ZRUA1361/20	2020	Mapulaneng Hospital	25	Male	Severe	Negative	Positive	Positive	≥1:32	Plasma/Serum
ZRUA1515/21	2021	Kalafong Provincial Tertiary Hospital	1	Female	Severe	Negative	Positive	Positive	≥1:32	Plasma/Serum
ZRUA1525/21	2021	Mapulaneng Hospital	53	Male	Severe	Negative	Positive	Positive	≥1:32	CSF
ZRUA1526/21	2021	Mapulaneng Hospital	37	Male	Severe	Negative	Positive	Positive	≥1:32	Plasma/Serum
ZRUA1530/21	2021	Matikwana Hospital	3	Female	Severe	Negative	Positive	Positive	≥1:32	Plasma/Serum
ZRUA1539/21	2021	Mapulaneng Hospital	39	Female	Moderate	Negative	Inconclusive	Positive	≥1:32	Plasma/Serum
ZRUA1544/21	2021	Mapulaneng Hospital	37	Male	Severe	Negative	Inconclusive	Positive	≥1:16	Plasma/Serum
ZRUA1547/21	2021	Mapulaneng Hospital	24	Male	Severe	Negative	Inconclusive	Positive	≥1:32	Plasma/Serum
ZRUA1552/21	2021	Mapulaneng Hospital	2	Female	Severe	Negative	Inconclusive	Positive	≥1:32	Plasma/Serum
ZRUA1601/21	2021	Mapulaneng Hospital	29	Female	Severe	Negative	Positive	Positive	≥1:16	Plasma/Serum
ZRUA1603/21	2021	Mapulaneng Hospital	25	Female	Moderate	Negative	Inconclusive	Positive	≥1:32	Plasma/Serum
ZRUA1566/21	2021	Mapulaneng Hospital	15	Female	Severe	Negative	Positive	Positive	≥1:32	Plasma/Serum
ZRUA1569/21	2021	Kalafong Provincial Tertiary Hospital	1	Male	Moderate	Negative	Positive	Positive	≥1:16	Plasma/Serum

Table S3. Demographic data of the AFDUC patients enrolled for West Nile virus molecular testing 2019 to 2021.

	Gauteng (n=646)		Mpumalanga (n=333)		Total (N=979)	
	**Frequency**	**Percentage (%) [95.00% CI]**	**Frequency**	**Percentage (%) [95.00% CI]**	**Frequency**	**Percentage [95.00% CI]**
**Age**						
Children (0-12 years)	505/646	78,17 [74,83 - 81,19]	193/333	57,96 [52,59 - 61,14]	698/979	71,30 [68,38 - 74,04]
Adolescents (13-18 years)	5/646	0,77 [0,33 - 1,80]	11/333	3,30 [1,85 -5,82]	16/979	1,63 [1,01 - 2,64]
Adults (19-59 years)	133/646	20,59 [17,65 - 23,88]	115/333	34,53 [29,63 - 39,79]	248/979	25,33 [22,71 - 28,15]
Senior adults (≥60 years)	3/646	0,46 [0,16 - 1,36]	14/333	4,20 [2,52 - 6,93]	17/979	1,74 [1,09 - 2,76]
**Sex**						
Male	362/646	56, 04 [52,19 - 59,82]	179/333	53,75 [48,39 - 59,04]	541/979	55,26 [52,13 - 58,35]
Female	283/646	43,81 [40,03 - 47,66]	151/333	45,35 [40,08 - 50,72]	434/979	44,33 [41,25 - 47,46
Unknown	1/646	0,15 [0,03 - 0,87]	3/333	0,90 [0,31 - 2,61]	4/979	0,41 [0,16 - 1,05]

Table footnotes: AFDUC = acute febrile disease of unknown cause, CI = confidence interval
